# Anticancer activities of ethanol extract from the Antarctic freshwater microalga, *Botryidiopsidaceae* sp.

**DOI:** 10.1186/s12906-017-1991-x

**Published:** 2017-12-01

**Authors:** Sung-Suk Suh, Sun-Mi Kim, Jung Eun Kim, Ju-Mi Hong, Sung Gu Lee, Ui Joung Youn, Se Jong Han, Il-Chan Kim, Sanghee Kim

**Affiliations:** 1 Division of Polar Life Sciences, Korea Polar Research Institute, Incheon, 21990 Republic of Korea; 2 Department of Polar Ocean Environment, Korea Polar Research Institute, Incheon, 21990 Republic of Korea; 3 Department of Polar Sciences, University of Science and Technology, Incheon, 21990 Republic of Korea; 4 Department of Pharmacy, Graduate School, Sungkyunkwan University, Suwan, 16419 Republic of Korea

**Keywords:** Antarctic freshwater microalga, *Botryidiopsidaceae* sp., Anticancer activities, Anti-proliferation

## Abstract

**Background:**

Cancer is a leading cause of human death around the world and occurs through the highly complex coordination of multiple cellular pathways. Recent studies have revealed that microalgal extracts exhibit considerable pharmaceutical activities, including those against various cancer cells. Thus, microalgae are promising candidates as novel cancer therapeutic drugs. In this study, we evaluated the biological functions of the ethanolic extract of the Antarctic freshwater microalga, *Bo* *tryidiopsidaceae* sp., such as its antioxidant, anti-proliferative, apoptotic and anti-invasive properties.

**Methods:**

To estimate antioxidant capacity of ethanol extract of *Bo* *tryidiopsidaceae* sp. (ETBO), free radical 2,2′-azino-bis (3-ethylbenzthiazoline-6-sulphonic acid) (ABTS) and 1,1-diphenyl-2-picrylhydrazyl (DPPH) assays were used. The anti-proliferative activity of ETBO was assessed in several cancer cell lines (A375, Hs578T and HeLa) and non-tumorigenic keratinocyte cells (HaCaT), using MTT assay. In addition, Annexin V binding was performed to detect ETBO-induced apoptotic cells, and the expression levels of apoptosis-regulating proteins, caspase-3, p53, and Bcl-2, were determined by western blot. Boyden chamber assays were used to determine anti-migratory and anti-invasive properties of ETBO.

**Results:**

ETBO exhibited antioxidant activity and concentration-dependent anticancer activities, such as anti-proliferation and pro-apoptotic activities against cancer cells. Furthermore, the expression of the apoptosis-inducing proteins, p53 and caspase-3, significantly increased in response to ETBO, whereas the expression of the anti-apoptotic protein, Bcl-2, decreased. These data imply that ETBO induces apoptosis by caspase activation through the modulation of pro-apoptotic and anti-apoptotic gene, p53 and Bcl-2, respectively. In addition, ETBO significantly inhibited migration and invasion of cervical cancer cells in a concentration-dependent manner.

**Conclusion:**

In this study, ETBO exhibited considerable anticancer activities, such as inhibition of proliferation, invasion, and migration, as well as induction of apoptosis. These data suggest that ETBO is a promising therapeutic agent in cancer therapy and drug discovery.

## Background

Over the past few decades, despite many promising treatments, targeted therapies that can selectively kill cancer cells have not yet been realized. For successful therapeutic treatment, a deep understanding of the specific metabolic characteristics distinguishing cancerous from normal cells, which can be targeted by therapeutic compounds, is required. For example, during tumorigenesis, cancer cells exhibit altered metabolic processes to provide energy and macromolecule precursors to maintain their abnormal rapid proliferation. In fact, the reduction of mitochondrial respiration, one of the dysregulated properties in cancer cells, prevents a complete conversion of glucose into carbon dioxide and water, leading to accumulation of a variety of precursors used by major biosynthetic pathways. Furthermore, this dysregulation of mitochondrial metabolism can cause a increase in reactive oxygen species (ROS) to induce DNA damage, causing uncontrolled rapid growth of cancer cells. Thus, the comprehensive understanding of the altered metabolism that is a hallmark in cancer cells could be necessary for the development of new anticancer treatments that selectively target oncogenic pathways in tumorigenesis [1–3].

In recent decades, many innovative anticancer drugs have been developed in the fight against cancer through the analytical validation of a variety of natural bioactive compounds [4, 5]. Furthermore, in drug development, these compounds have renewed interest in alternative sources of effective therapeutics due to the limitations of delivery of many bioactive compounds. These natural compounds may be used as templates for the development of new drugs by the pharmaceutical industry. According to the 2015 annual report of the American Association for Cancer Research (AACR), more than 800 medicines and vaccines had been developed for cancer therapeutic treatments that were in active clinical trials. Interestingly, of these drugs, approximately 40% have their origins in natural products derived from plants, animals and microorganisms, or their semisynthetic derivatives. In recent studies, their pharmaceutical importance as sources of new therapeutic agents against human diseases including cancer, hypertension, infective, immunosuppression, and neurological disease therapeutic areas has been emphasized [6–10].

Microalgae, single-celled photosynthetic eukaryotes, are widely distributed in the world. Their evolutionary adaptation to a wide range of habitats and extreme environments has allowed microalgae to have an abundance of biological and genetic diversity, potentially producing a variety of bioactive compounds. In fact, it has been recently reported that several algae-derived bioactive metabolites exhibit health-promoting activities, and their pharmacological values attract attention in the development of new drugs [11–13]. In particular, some compounds exhibit pharmacological activity by regulating multiple biological processes, such as cell proliferation, metastasis and apoptosis in cancer cells [14, 15]. For examples, fucoxanthin derived from marine organisms such as microalgae, macroalgae and seaweeds, potentially functions as anticancer agent by modulating apoptotic signaling and inducing cell cycle arrest [16, 17].

Recently, numerous bioactive metabolites from organisms which live in the extreme environments, including the Antarctic region, have gained increasing attention from pharmaceutical industry. These organisms can synthesis essential secondary metabolites that are necessary for the survival in harsh conditions, and these metabolites can be explored as therapeutic treatments for human diseases, including cancer [7, 18]. In this study, we aimed to investigate anticancer activities of ethanolic extract derived from *Botryidiopsidaceae* sp., an Antarctic freshwater microalga, to determine whether ETBO contains potential pharmaceutical compounds with anticancer activities.

## Methods

### Sample preparation

The microalga, *Botryidiopsidaceae* sp., was obtained near King Sejong Station (62° 13′ S, 58° 47′ W). The sequences of 18S rDNA in the nuclear genome were amplified by PCR, cloned and sequenced. The clone sequences were analyzed by BLAST for sequence similarities with the NCBI GenBank database. The microalga, *Botryidiopsidaceae* sp., was deposited in Korea Polar Research Institute, Republic of Korea (# KSF211). Twelve gram of dried microalgal material was used for ethanol extraction, as described in previous work [19].

### Free radical scavenging assay

The DPPH and ABST radical methods [19] was modified to evaluate the free radical-scavenging of ETBO. Briefly, in the DPPH assay, DPPH (8 mg) was dissolved in methanol (100 mL) for a stock solution of 80 μg/mL. To prepare working DPPH reagent, its stock solution was diluted with methanol until the absorbance at 514 nm was 0.650 ± 0.020. Then, 2.95 mL of the working solution was mixed with 50 μL of sample. After incubation in the dark at room temperature for 20 min, the absorbance was measured at 514 nm. In the ABTS assay, to prepare ABTS reagent, 5 mL of a stock solution (7 mM) was mixed with 88 μL of 140 mM potassium persulfate. The mixture was maintained at room temperature for 4–16 h until radical generation was completed and the absorbance was stable. To determine the scavenging activity, 0.1 mL of each tested samples was mixed with 0.9 mL of ABTS∙^+^Solution for 1 min, and its absorbance was measured at 734 nm after incubation for 10 min. The percent inhibition of DPPH or ABTS free radicals was determined using the following formula:

$$ \%\mathrm{inhibition}=\left\lfloor \frac{\left(\mathrm{A}\right)\mathrm{control}-\left(\mathrm{A}\right)\mathrm{sample}}{\left(\mathrm{A}\right)\mathrm{control}}\right\rfloor \times 100. $$

Where ‘(A) control’ was the absorbance of the control and ‘(A) sample’ was the absorbance after ETBO treatment. Ascorbic acid was used as a reference standard compound. The IC_50_ value, which is the concentration that can inhibit 50% of DPPH or ABST free radicals, was obtained by extrapolation from regression analysis.

### Cell culture and proliferation assay

All cell lines used in this study were obtained from the American Type Culture Collection (ATCC, VA, USA) and were grown in Dulbecco’s modified Eagle medium (DMEM) containing 10% fetal bovine serum (FBS) and 1% penicillin-streptomycin. Cells were seeded in a 96-well plate in triplicate at a density of 5×10^3^ cells per well and incubated at 37 °C in a humidified 5% CO_2_ incubator. After overnight incubation, the cells were treated with different concentrations (0.8–50 μg/ml) of ETBO for 24 h. A negative control without the ETBO was used, and the final volume of each well was adjusted to 200 μl with growth media. Cell proliferation was measured for 72 h. At every 24 h interval, 20 μl MTT (3-(4,5-dimethylthiazol-2-yl)-2,5-diphenyltetrazolium bromide, Promega, USA) was added into a subset of wells. After 1 h incubation, the absorbance was measured in a Multilabel Counter (Bio-Rad Laboratories, USA). In addition, changes in cell morphology were observed under a phase-contrast inverted microscope (Nikon D700).

### Apoptosis assay

Using a flow cytometric assay system (BD Biosciences, San Jose, CA, USA), Annexin V–fluorescein isothiocyanat (FITC) binding to apoptotic cells was measured. The ETBO-treated cells were washed twice with cold phosphate-buffered saline (PBS) and resuspended in 1 × binding buffer (BD Biosciences, San Jose, CA, USA) at a concentration of ~1 × 10^6^ cells/mL. 100 microliters of the suspension (~1 × 10^5^ cells/mL) was transferred to a 5 ml culture tube, and Annexin V-FITC (5 μl) and propidium iodide (PI, 2 μl) solution were added to each tube and gently mixed with the cells by pipetting, respectively. After incubation at room temperature for 20 min, flow cytometry assay was performed to evaluate apoptotic cells in response to ETBO.

### Western blot analysis

Cells were lysed with RIPA buffer (Sigma-Aldrich, St. Louis, MO, USA) and constantly agitated for 30 min. The cell lysate was centrifuged in a microcentrifuge at 4 °C, and the supernatant was collected in a fresh tube kept on ice. Equal amount of total protein (30 μg) was used for the western blot detection of each target gene [19]. The primary antibodies used detected caspase-3, Bcl-2, and p53 (Cell Signaling, USA). After probing with secondary antibody conjugated to horseradish peroxidase (HRP, Santa Cruz Biotechnology, USA), the protein signals were detected using film and chemiluminescence (Amersham Pharmacia, USA).

### Colony forming assay

Cells were seeded at an initial density of 1 × 10^3^ cells/well in a 6-well plate. After overnight incubation for cell adherence, the cells were treated with different doses of ETBO (12.5 and 25 μg/ml) and then incubated until colonies were detectable. Cells were fixed, stained, and counted as described in our previous study [19].

### Invasion and migration assays

Using 24-well migration and invasion plate containing polycarbonate membrane inserts (Cell Biolabs, USA), the migratory and invasive capacity of cancer cells was measured in response to ETBO. The cell suspension solution (300 μl) containing 1.0 × 10^6^ cells/ml in serum-free media was added to the top chamber and treated directly with different concentrations of ETBO (1.6 and 3.2 μg/mL). The bottom chamber contained media with 10% FBS media (500 μl). After 48 h incubation, the cells that migrated or invaded were stained using Cell Stain Solution (400 μl) and photographed.

### Statistical analysis

The results represent the mean ± SD values for three independent biological experiments. Statistical analysis was performed with SigmaPlot software (Systat Software, Inc., CA, USA), using Student’s t test; *p* values <0.05 indicated a statistically significant difference.

## Results

### Antioxidant activity of ETBO

**Fig. 1:**
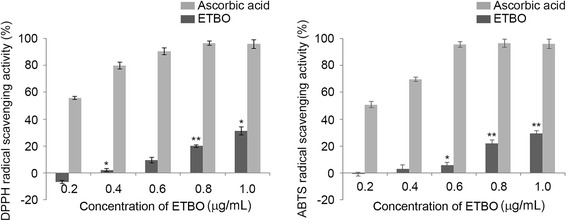
Antioxidant activities of ETBO: DPPH and ABTS assays. Quantitation of the results from three independent experiments (*n* = 3) is shown as the mean ± SD. *p < 0.05 and **p < 0.01 between the control and ETBO-treated group. ETBO, Ethanol extract of *Botryidiopsidaceae* sp.; DPPH, 1,1-diphenyl-2-picrylhydrazyl; ABTS, 2,2′-azino-bis (3-ethylbenzthiazoline-6-sulfonic acid)

There is accumulating evidence that cellular damage caused by reactive oxygen species (ROS) is one cause of aging, leading to age-related diseases such as Alzheimer’s disease and cancer [20, 21]. Thus, antioxidants have received considerable attention due to their capability to attenuate deleterious effects of ROS. In the present study, to determine the free radical scavenging capacity of ETBO, we preformed both ABTS and DPPH assays, which are extensively used in spectrophotometric systems to determine the scavenging activities of extracts derived from plant, animal and microorganism. In the DPPH assay, the percent inhibition of ETBO increased in a concentration-dependent manner, showing a range of 1.51–32.15% with an IC_50_ value of 1.53 μg/mL (Fig. 1); the positive control, ascorbic acid, had an IC_50_ value of 0.15 μg/mL. In addition, in the ABTS assay, the percent inhibition ranged between 2.15% at 0.4 μg/mL and 27.84% at 1.0 μg/mL comparing with the ascorbic acid (97.25%). ETBO exhibited IC_50_ value of 1.79 μg/mL, whereas the IC_50_ of standard compound, ascorbic acid was 0.19 μg/mL.

### ETBO-mediated inhibition of cell proliferation in human cancer cells

**Fig. 2:**
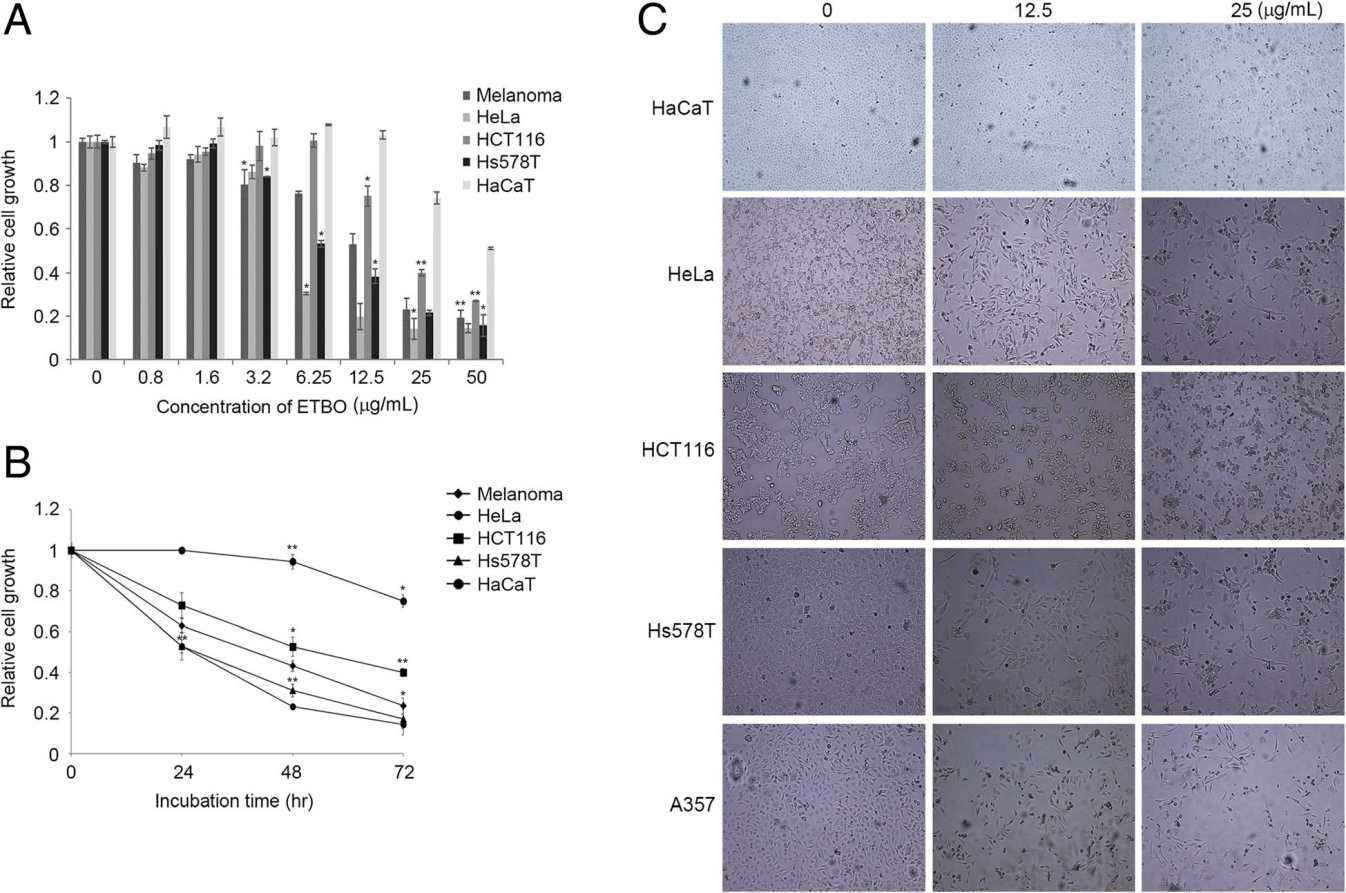
Antiproliferative effects and morphological changes of human cancer cells treated with ETBO. Antiproliferative activity of ETBO was measured in four different cancer cell lines treated with its different concentrations (0 - 50 μg/mL) for 24 h (**a**) and treated with 12.5 μg/mL of ETBO for 72 h (**b**). Morphological changes were observed in the cancer cells treated with or without different concentrations (12.5 and 25 μg/mL) of ETBO for 72 h (**c**). The cell viability was assessed by MTT assay. The results are shown as the means ± SD, *n* = 3. *p < 0.01, **p < 0.001 between the control and ETBO-treated group. ETBO, Ethanol extract of *Botryidiopsidaceae* sp.; MTT, 3-(4,5-dimethylthiazol-2-yl)-2,5-diphenyltetrazolium bromide

**Fig. 3:**
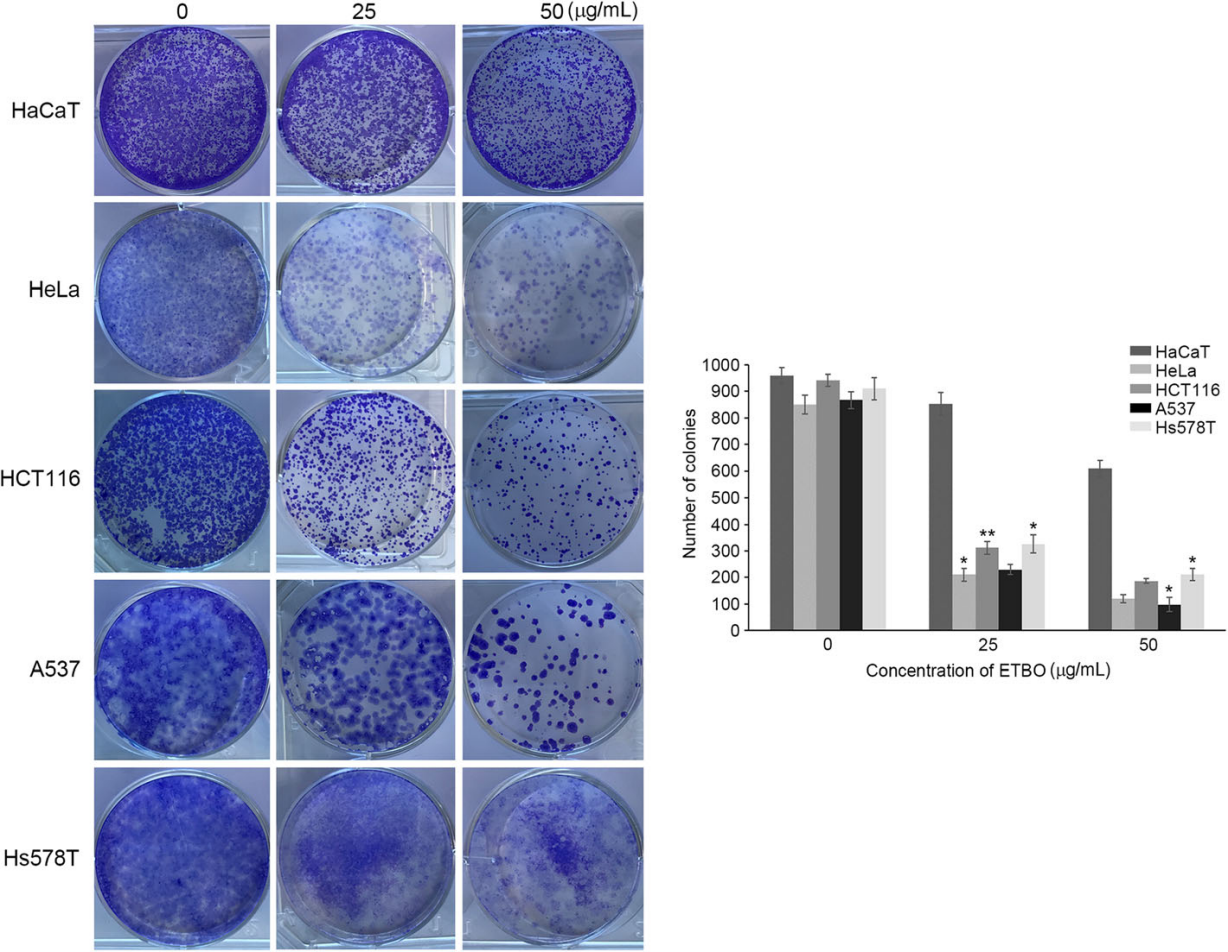
Inhibition of colony formation of cancer cells after ETBO (25 and 50 μg/mL) treatment for 12 h. Experiments were performed three times and the data are presented as the mean ± SD. *p < 0.05, **p < 0.01 between the control and ETBO-treated group

To evaluate the therapeutic potentials of ETBO against human cancer, four cancer cell lines (HeLa, HCT116, Hs678T and A537) were treated with different concentrations of ETBO (0, 0.8, 1.6, 3.2, 6.25, 12.5, 25, 50 μg/mL) for 24 h. As shown in Fig. 2a, cell proliferation in ETBO-treated HeLa and Hs578T was concentration-dependently inhibited at ≥6.25 μg/mL, whereas suppression of proliferation of Hs578T and A375 was observed at ≥12.5 μg/mL. In contrast, we observed that there was no significant inhibition of cell growth in HaCaT cells that were derived from normal human skin. This result implies that ETBO exhibits selective cytotoxicity to at least different cancer cell lines. In addition, we observed that the cellular viability of all tested cancer cell lines was significantly decreased in a time-dependent manner after the treatment with ETBO (12.5 μg/mL), compared with that of the normal cells (Fig. 2b). In particular, HeLa was more sensitive to ETBO treatment than the other cancer cell lines were. Based on these data, ETBO may exhibit a broad inhibitory spectrum against cancer cells in a concentration- and time-dependent manner. Furthermore, the data was supported by the morphological observations of the ETBO-treated cancer cells, showing that visible changes in cell morphology indicative of cell death, such as shrinking (Fig. 2c). In addition, consistent with the cell proliferation assay, the number of cancer cell colonies markedly reduced in response to ETBO (25 and 50 μg/mL) compared to that of normal cells (Fig. 3).

### ETBO-inducted apoptosis in human cancer cells

**Fig. 4:**
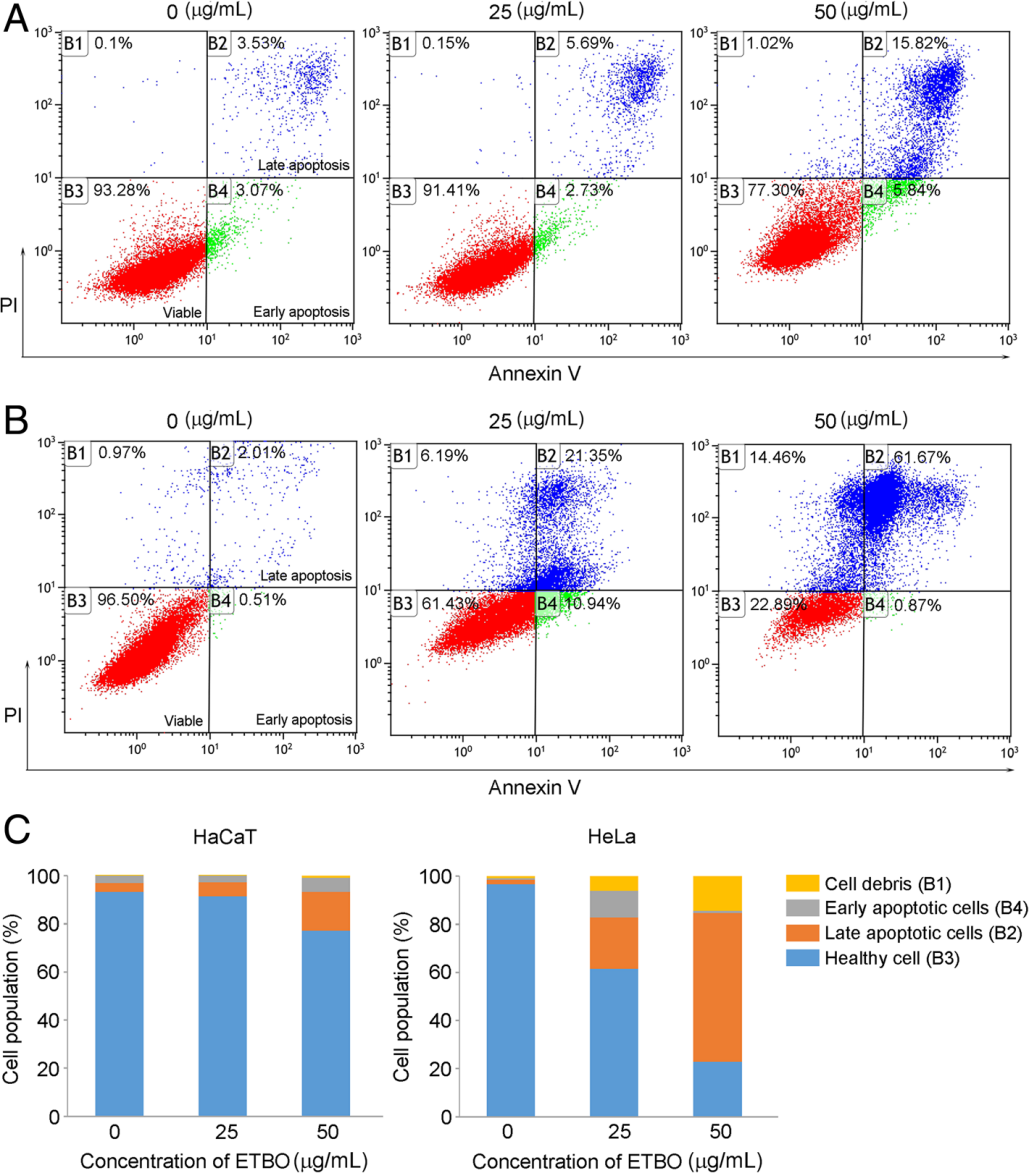
Effect of ETBO on apoptosis. Normal (HaCaT) (**a**) and cancer (HeLa) cells (**b**) were treated with or without different concentrations (25 and 50 μg/mL) of ETBO for 24 h. Cell population and the extent of apoptosis were measured by FACS analysis (**c**). ETBO, Ethanol extract of *Botryidiopsidaceae* sp.; FACS, fluorescence-activated cell sorting

**Fig. 5:**
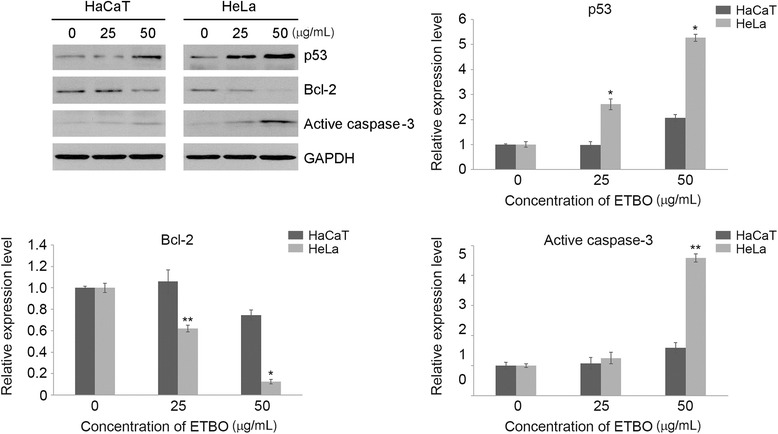
Western blot analysis for apoptosis-related proteins, caspase-3, Bcl-2, and p53. The results are the means ± SD, n = 3. *p < 0.05, **p < 0.01 between the control and ETBO-treated group

Next, we performed flow cytometry to evaluate the apoptotic effect of ETBO on cancer cells. In response to ETBO treatment, significant apoptosis occurred in HeLa cells with increasing doses of ETBO: 2.52% (0 μg/mL), 32.29% (25 μg/mL) and 62.54% (50 μg/mL), compared to that of HaCaT cells: 6.60% (0 μg/mL), 8.42% (25 μg/mL) and 21.66% (50 μg/mL) (Fig. 4). To further understand the molecular mechanisms underlying ETBO-induced apoptosis, western blotting was performed to investigate the expression levels of apoptotic markers Bcl-2, caspase-3, and p53 in human cancer and normal cells in response to ETBO treatment for 24 h. Consistent with the flow cytometry data, expression of anti-apoptotic Bcl-2 protein was remarkably decreased in a concentration-dependent manner, whereas apoptosis-inducing genes, caspase-3 and p53, were increased (Fig. 5). Taken together, our data suggest that ETBO induces or enhances apoptosis in cancer cells by activating caspase-3 via apoptotic pathway.

### Inhibition of cell invasion and migration by ETBO

**Fig. 6:**
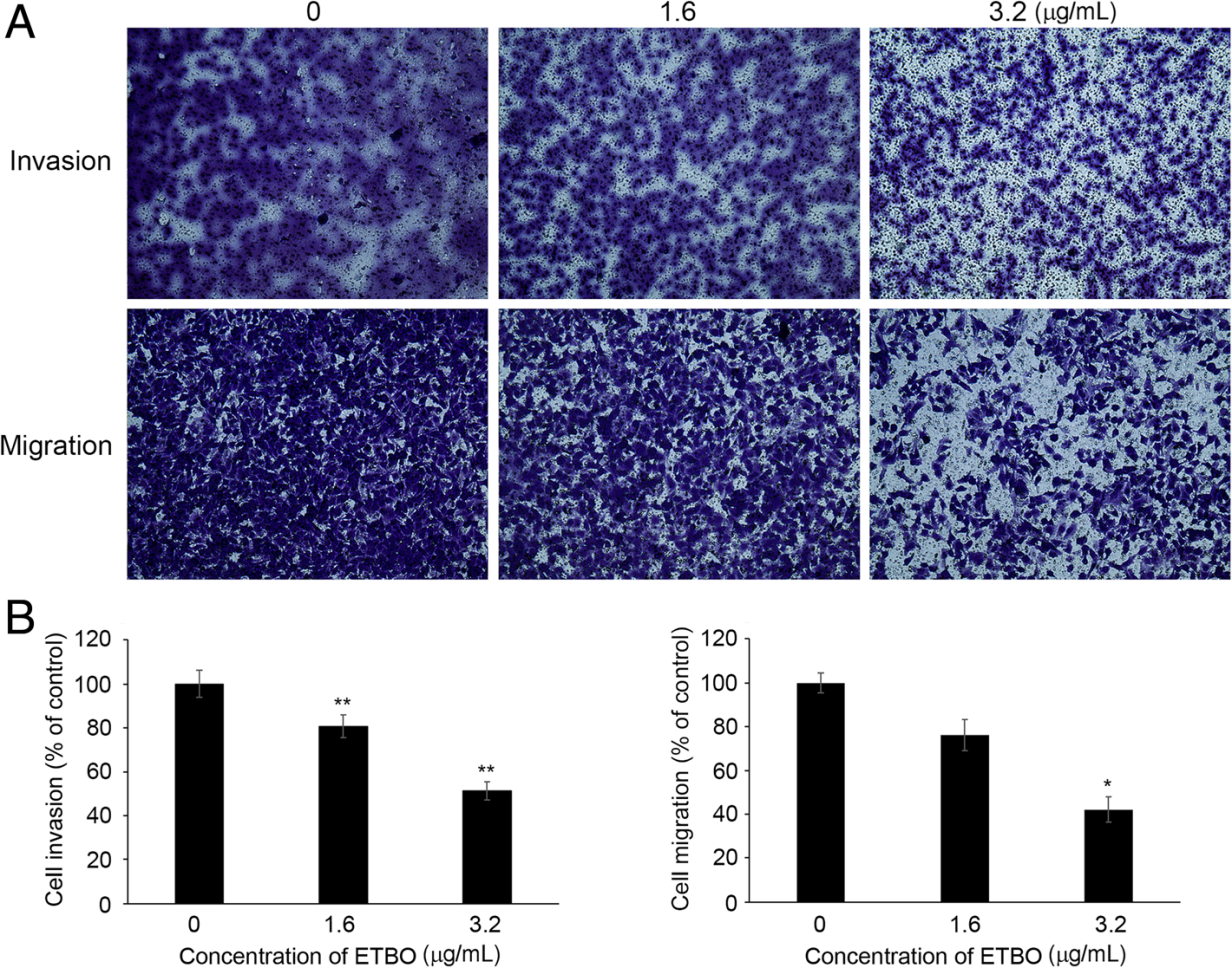
Effect of ETBO on cellular invasion and migration. Cells (HeLa) were treated with or without different concentrations (1.6 and 3.2 μg/mL) of ETBO for 24 h (**a**). The quantitation of the results from three independent experiments (n = 3) is shown as the mean ± SD with statistical significance as *p < 0.05 and **p < 0.01 between the control and ETBO-treated group (**b**). ETBO, Ethanol extract of *Botryidiopsidaceae* sp.

Cell invasion and migration have been demonstrated to play a crucial role in cancer metastasis, which is the primary cause of cancer mortality, leading to extremely poor diagnosis and survival in patients [22]. To study the role of ETBO in the metastatic processes of invasion and migration, Boyden chamber assay was performed using 1.6 and 3.2 μg/mL, which did not affect the proliferation of HeLa cells (Fig. 1). As shown in Fig. 6, ETBO significantly suppressed the invasion and migration capacity of cancer cells in a concentration-dependent manner. Notably, ETBO-mediated inhibition of cancer cell invasion and migration was not due to a cytotoxic effect of ETBO. These data indicated that the invasion and migration capacities through the basement membrane were significantly suppressed in ETBO-treated cancer cells, compared with those of the control cells, suggesting that ETBO can suppress cancer invasion and migration.

## Discussion

In the present study, we investigated the anticancer activity of ETBO from an Antarctic freshwater microalga, *Botryidiopsidaceae* sp., in human cancer cell lines including HeLa, HCT116, Hs678T and A537. Based on our data, ETBO significantly inhibited cancer cell growth and induced cellular apoptosis through the modulation of apoptotic genes such as p53, Bcl-2, and caspase-3 gene. In general, apoptosis occurs through the comprehensive interaction of a variety of apoptotic regulator proteins, such as Bcl-2, caspase, and p53. For examples, Bcl-2 plays a crucial role in the regulation of cell death and cell proliferation through the inhibition of apoptosis, preventing the activation of pro-apoptotic caspase proteins such as caspase-3/7 [23]. In addition, p53, a tumor suppressor gene, functions as a negative regulator of Bcl-2 at the transcriptional level [24]. In fact, dysregulation in the apoptotic pathway has been thought to contribute to malignant transformation [25]. Thus, targeted induction of apoptosis is an efficient strategy in cancer therapy and drug development. ETBO has profound cytotoxic effects against human cancer cells through the activation of pro-apoptotic signaling in response to ETBO treatment (Fig. 5). It is known that there are two pathways, extrinsic and intrinsic pathways, which can transmit cellular apoptotic signals to the apoptotic regulatory system. The “extrinsic” pathway initially occurs via death receptors to bind their ligands and activating the caspase cascade [26]. The activated caspase cascade can induce the release of mitochondrial cytochrome c into the cytoplasm, which also leads to the activation of effector caspases. In contrast, “intrinsic” pathway is directly triggered by numerous apoptotic signals that facilitate cytochrome c release [26]. In general, apoptotic pathway consists of sequential biochemical events, including the activation of caspases [26], a family of cysteine protease that plays crucial roles in apoptotic signaling. Caspases can be divided into initiator (caspase-8 and -9) and executioner caspases (caspase-3 and -7). Initiators caspase-8 and -9 can be activated through the extrinsic pathway and mitochondrial cytochrome c leakage, respectively. Both initiator caspases can activate executioner caspase, caspase-3 or −7, which are mainly responsible for the final stages of apoptosis [26]. Thus, based on our data showing caspase-3 activation, ETBO may promote apoptosis in cancer cells via extrinsic or mitochondrial-dependent intrinsic pathways. In addition, other essential regulatory proteins for apoptotic signaling include Bcl-2 and p53; both proteins are major regulators of cell survival or cell death. Bcl-2 functions as an inhibitor of apoptosis and an oncogene in tumor progression [27], whereas p53 plays a vital role in tumor suppression via the modulation of multiple biological processes related to anti-proliferative activities, such as cell cycle and apoptosis [28]. Our data show that the expression levels of Bcl-2 significantly reduced in response to ETBO treatment, implying the significance of Bcl-2 proteins for cancer cell survival. It has been noted that Bcl-2 plays a crucial role in protecting mitochondria from cellular malfunctions that occur during apoptosis [25]. Indeed, cell death is significantly prevented by anti-apoptotic genes, including Bcl-2, through inhibition of the apoptosis-mediated release of mitochondrial cytochrome c into the cytoplasm [23–25]. In addition, many studies have demonstrated that p53 plays a central role in the transmission of various death signals to both the extrinsic and intrinsic apoptotic pathways [26]. Therefore, downregulation or upregulation of Bcl-2 or p53, respectively, in ETBO-treated cancer cells could promote the release of mitochondrial cytochrome c and activate the caspase cascade; p53 activation can trigger cytochrome c release into the cytoplasm via the Bcl-2-mediated pathway, and the released cytochrome c activates caspase-3. Our data suggest that ETBO induces or enhances apoptosis in cancer cells by activating caspase-3 via extrinsic and intrinsic apoptotic pathways. On the other hand, metastasis, the leading cause of cancer mortality, occurs via numerous sequential events, including cell invasion and migration under the sophisticated control of molecular regulatory systems, including metastasis regulatory genes [22]. In this study, we observed that ETBO significantly suppressed cell invasion and migration (Fig. 6), suggesting that the inhibitory effects of ETBO on migration and invasion may occur via the modulation of genes related to the processes of cellular invasion and migration. Further experiments examining this possibility are warranted.

## Conclusions

This study showed that ETBO from an Antarctic microalga, *Botryidiopsidaceae* sp. has profound anticancer activity in various human cancer cell lines. Based on our data, Antarctic microalgal extract significantly inhibits the proliferation of cancer cells through induction of apoptosis by the regulation of major apoptosis-related proteins, such as caspase-3, p53 and Bcl-2. In addition, cell invasive and migratory capacity of cancer cells was considerably suppressed in response to ETBO treatment. Further studies are required to evaluate ETBO therapeutic effects on other cancer cell lines or in vivo xenograft model and to identify the bioactive components responsible for the anti-cancer property of ETBO. These findings will help promote therapeutic application of the microalgal extract and its constitutes on human cancers.

## Abbreviations

ABTS: 2,2′-azino-bis(3-ethylbenzthiazoline-6-sulfonic acid)
ATCC: American Type Culture Collection
DMEM: Dulbecco’s modified Eagle’s medium
DPPH: 1,1-diphenyl-2-picrylhydrazyl
ETBO: Ethanol extract of *Botryidiopsidaceae* sp.
FBS: Fetal bovine serum
FITC: Fluorescein isothiocyanate
HRP: Horseradish peroxidase
IC_50_: Half maximal inhibitory concentration
MTT: 3-(4,5-dimethylthiazol-2-yl)-2,5-diphenyltetrazolium bromide
PBS: Phosphate-buffered saline
PI: Propidium iodide
ROS: Reactive oxygen species
